# Inductive flash-annealing of bulk metallic glasses

**DOI:** 10.1038/s41598-017-02376-x

**Published:** 2017-05-19

**Authors:** K. Kosiba, S. Pauly

**Affiliations:** IFW Dresden, Institut für Komplexe Materialien, Helmholtzstraße 20, D-01069 Dresden, Germany

## Abstract

We developed a temperature-controlled inductive flash-annealing device, which heats bulk metallic glasses (BMGs) at defined rates of up to 200 K/s to a given temperature. Subsequent instantaneous quenching in water allows preserving the microstructures obtained at various stages of crystallization. One Zr-based and two CuZr-based BMGs were flash-annealed at the onset of crystallization with different heating rates in order to prepare advanced BMG-matrix composites. The highly reproducible composite microstructures contain uniformly dispersed crystals and a narrow crystal size distribution. In order to assess the limitations of the present process, which mainly originate from non-uniform inductive heating, the skin depth was calculated. It is determined to be about 2.3 mm, which enables flash-annealing of rather bulky samples. The cooling rate was estimated from the interlamellar spacing of eutectic Al-Cu alloys to be on the order of 10^3^ K/s. This ensures that decomposition of the microstructure during quenching is prevented. The present flash-annealing procedure is applicable to a wide variety of glass-forming liquids and has a large potential for tailoring the microstructure and, consequently, the mechanical properties of BMG-matrix composites.

## Introduction

Structurally disordered materials like metallic glasses are poor electrical conductors in comparison to their crystalline counterparts^1, 2^. This is the reason why metallic glasses can be heated extremely fast by ohmic heating, even up to a point where the supercooled liquid transforms to a stable liquid without crystallizing in between^3^. When quenching succeeds such rapid heating, the resulting annealing procedure is also known as flash-annealing^4–7^. Flash-annealing has been originally performed on amorphous Fe- and Co-based ribbons to improve their magnetic properties^4, 5, 7–9^. These glass-forming alloys have been heated to above their glass-transition temperature, *T*_g_, for very short durations^8, 9^. The subsequent cooling, however, has occurred at rates orders of magnitude below the cooling rate typical of melt spinning^10^. Residual stresses in the melt-spun ribbons can thus be relieved and the magneto-elastic anisotropy of the glasses is reduced^7, 9^. After flash-annealing, the structurally relaxed samples show a lower coercive field compared to the as-cast state and hence are more soft-magnetic^7, 9^. This relaxation treatment, however, is only effective in some cases^7, 11^ when the precipitation of crystalline phases is suppressed.

Full or partial devitrification of a supercooled liquid has to be also avoided during thermoplastic forming of metallic glasses^3, 12, 13^. In this case, ultrafast heating of metallic glasses to a temperature regime in which the viscosity is ideal for forming (10^2^–10^4^ Pa·s)^3, 14^, is a promising approach. The time required for the entire process of thermoplastic forming can be significantly shorter than the typical time scale of crystallization so that even marginal glass formers can be processed^3^. Thermoplastic forming of BMGs during rapid heating was first proposed by Saotome *et al*.^12, 13^, who have micro-formed La- and Pt-based BMGs in the supercooled liquid region, i.e. between *T*_g_ and the onset of crystallization, *T*_x_. They have heated these BMGs by means of electromagnetic induction at rates ranging from 1 to 10^3^ K/s. This idea has been advanced more recently by heating bulk metallic glasses through millisecond capacitive discharge^3, 14, 15^. The maximum accessible heating rates reach up to 10^6^ K/s and this approach has the additional advantage that it results in spatially uniform heating over the entire temperature range^3, 14–16^. By contrast, inductive heating suffers from the skin effect, which generally leads to a temperature gradient –especially in large samples^17^.

Beyond that, flash-annealing proves to be a very promising tool in order to prepare very uniform BMG-matrix composites with adjustable crystal sizes^18^. On quenching of the melt, the maximum crystal growth rates are necessarily passed^19^. Crystal growth is slow at small undercoolings because of a limited thermodynamic driving force for crystallization and it is slow again at large undercoolings due to a low atomic mobility^20^. At intermediate undercoolings, the growth rate attains its maximum and its value can be relatively high^21^. Few but relatively large crystals precipitate on cooling of the melt in most glass-forming alloys as a consequence^22–26^. If a glass is heated instead, the annealing temperature can be chosen in such a way that the crystal growth velocity is below its maximum. Simultaneously, the annealing time has to be minimized in order to avoid excessive growth of the crystals. Both requirements are met when metallic glasses are flash-annealed, as we could recently demonstrate for Cu_44_Zr_44_Al_8_Hf_2_Co_2_^18^, one of the alloys investigated in more depth in this work.

When crystallization occurs under conditions far from equilibrium, it can be severely affected in terms of phase formation as well as the nucleation process. Several studies on the crystallization of Fe-, Zr- and CuZr-based BMGs^6, 18, 27^ report an influence of the applied heating rate on the phase formation. Metastable phases can be obtained and equilibrium phases might be suppressed during very fast heating due to kinetic constraints^6, 18, 27, 28^. In addition, flash-annealing annealing of a CuZr-based glass has revealed transient nucleation to become important on very fast devitrification^18^ and these experiments eventually verify the theoretical concept of heating rate-dependent nucleation rates^29^. Because the number of crystals and their size can be controlled almost independently by modifying the heating rate and the annealing temperature/time, flash-annealing provides access to BMG-matrix composites with adjustable crystalline volume fractions and interparticle spacing. Next to the morphology of the second phase, these microstructural features determine the deformation behaviour of the composites^30, 31^. Such a designing of composite microstructures is crucial for better understanding the deformation mechanisms and for optimizing the mechanical properties. Flash-annealing is hence capable of producing novel metastable microstructures, which cannot be synthesized otherwise.

The preparation of BMG-matrix composites critically depends on the details of the flash-anneal, i.e. the heating rate, annealing temperature, annealing duration and cooling rate. All these aspects have to be understood in order to control the flash-annealing process as well as the resulting composite microstructures properly. Assessing the technological capabilities of our flash-annealing setup is the aim of the present work. The bulk metallic glasses Cu_46_Zr_46_Al_8_, Cu_44_Zr_44_Al_8_Hf_2_Co_2_, and Zr_52.5_Cu_17.9_Ni_14.6_Al_10_Ti_5_ were flash-annealed at temperatures slightly above the onset of crystallization and immediately quenched in a custom-made device. Since the heating occurs via electromagnetic induction, the inherent skin effect could impair uniform heating. Based on the skin depth, we can estimate the maximum sample diameter, which still allows for uniform heating. The cooling rate of the process is inferred from the interlamellar spacing of flash-annealed eutectic Al_82.7_Cu_17.3_ specimens. Based on a systematic microstructural analysis, we can prove that the composite microstructure uniformly stretches across the entire sample volume chosen in the present work.

## Results and Discussion

**Figure 1:**
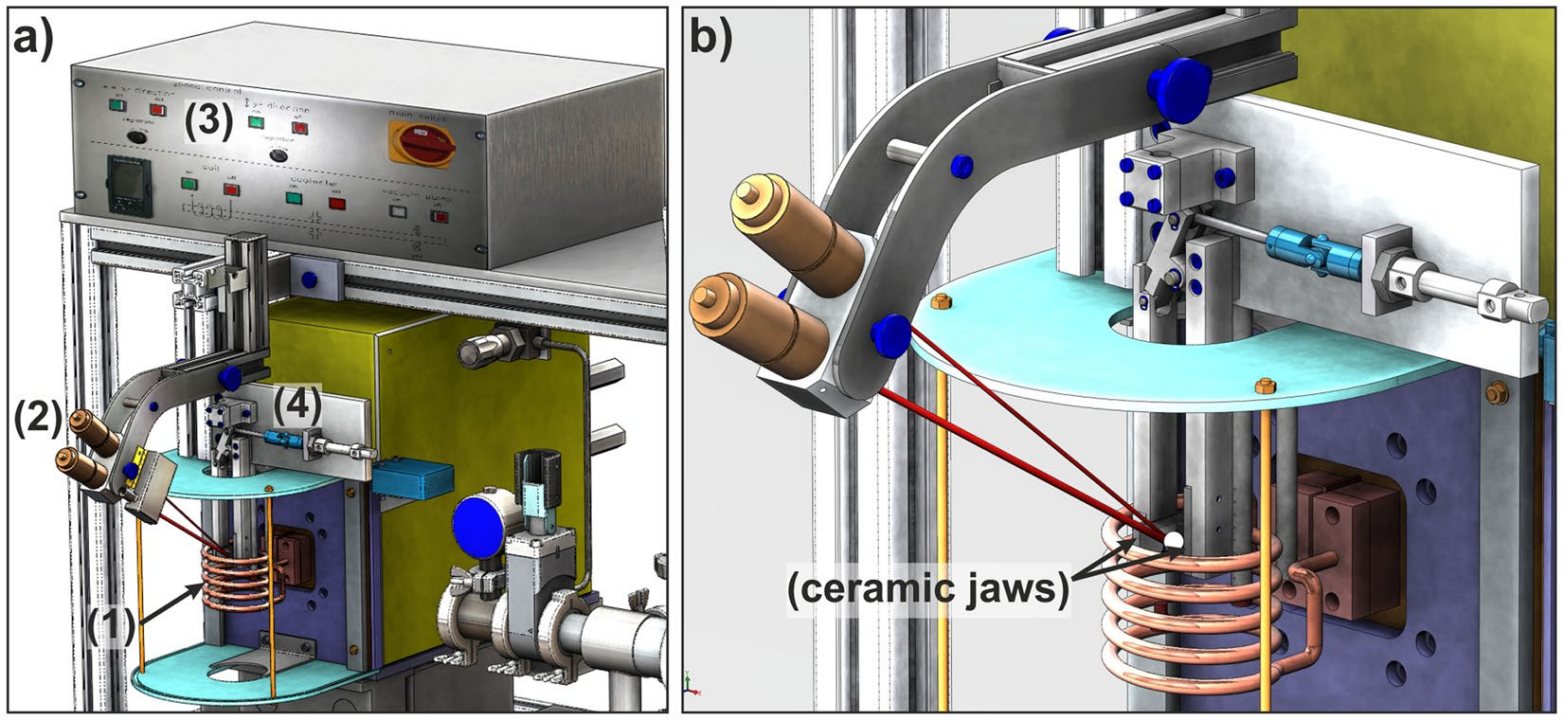
Setup of the flash-annealing device. (**a**) The BMG specimen is located within the water-cooled induction coil (1) and a high-speed pyrometer (2) records its surface temperature. When a pre-defined temperature is reached, the control unit (3) activates the clamping system (4), so that the specimen falls into a water-bath (not shown here), which is placed inside the coil directly below the specimen. (**b**) Magnification of the induction coil including the clamping system. The sample is located between two ceramic jaws (indicated by black arrows) and a white spot marks its position.

The present flash-annealing device is illustrated in Fig. 1a. It comprises a water-cooled-induction coil, a medium-frequency generator, a high-speed pyrometer, a control unit and a clamping system. The BMG specimen is located within the coil (Fig. 1b: white spot) between two ceramic jaws, which are attached to polymer beams. As the specimen is flash-annealed, the pyrometer monitors its surface temperature, which is then processed by the control unit. Once a preset temperature is reached, the control unit activates the clamping system, the specimen falls into a subjacent water-bath (not shown in Fig. 1) and is quenched.

It is known that capacitive discharge heating enables spatially uniform heating even at elevated temperatures and during fast heating^3, 14–16^, whereas inductive heating tends to heat the sample surfaces faster than the interior of an electrical conductor^14, 17, 32^. We are interested in uniform heating in order to obtain homogeneous BMG-matrix composite microstructures. The skin depth represents a critical quantity in this respect^14, 16^ and the sample radius should not exceed it significantly. Therefore, we start by calculating the skin depth for the present setup. The alternating electromagnetic field of the coil induces so-called eddy currents in the cross-section of the BMG specimens^33, 34^. The current density is largest near the surface and decreases exponentially towards the sample centre. Hence, the electric charge mainly flows at the surface of the sample because of the mutual repulsion of the electrons^17, 35, 36^. This phenomenon is called skin effect and describes a non-uniform current distribution over the cross-section. The skin depth, *s*, is defined as the distance from the sample surface at which the current density has decreased to *1/e* (36,7%) of its maximum value at the surface^17, 35, 36^. In the following, the equation for *s* for the present experimental setup, shall be deduced starting from the macroscopic Maxwell equations^17^:

1 $$rot\,\,\mathop{E}\limits^{\rightharpoonup }+\frac{\partial \mathop{B}\limits^{\rightharpoonup }}{\partial t}=0,$$

2 $$rot\,\,\mathop{H}\limits^{\rightharpoonup }+\frac{\partial \mathop{D}\limits^{\rightharpoonup }}{\partial t}=\mathop{j}\limits^{\rightharpoonup }$$

and

3 $$div\,\,\mathop{B}\limits^{\rightharpoonup }=0,$$

where $\mathop{E}\limits^{\rightharpoonup }$ is the electric field density, $\mathop{B}\limits^{\rightharpoonup }$ is the magnetic flux density, $\mathop{H}\limits^{\rightharpoonup }$ is the magnetic field strength, $\mathop{D}\limits^{\rightharpoonup }$ is the electric flux density and *t* is the time. The displacement current, $\frac{\partial \mathop{D}\limits^{\rightharpoonup }}{\partial t}$, can be neglected since the inductive heating takes place in the medium-frequency range^32^, and metallic glass is electrically conductive, so that (2) reduces to:

4 $$rot\,\,\mathop{H}\limits^{\rightharpoonup }\cong \mathop{j}\limits^{\rightharpoonup }.$$

The constitutive relation between magnetic flux density and magnetic field strength is given by ref. 32:

5 $$\mathop{B}\limits^{\rightharpoonup }={\mu }_{r}{\mu }_{0}\mathop{H}\limits^{\rightharpoonup },$$

where *μ*_r_ is the relative permeability and *μ*_0_ is the permeability of vacuum. The BMG is homogeneous and isotropic, so that its electrical conductivity, *σ*, can be approximated as a scalar:

6 $$\mathop{j}\limits^{\rightharpoonup }=\sigma \mathop{E}\limits^{\rightharpoonup },$$

By combining equations (1) to (6) one can obtain the following differential equation, which describes the partial and temporal distribution of the magnetic field strength as well as the electrical current density^32^:

7 $$\frac{1}{\sigma }{\rm{\Delta }}H=\frac{\partial H}{\partial t}{\mu }_{r}{\mu }_{0},$$

which converts to:

8 $$\frac{\partial {H}_{y}}{\partial {x}^{2}}=\frac{2i}{{s}^{2}}{H}_{y},$$

with:

9 $$s=\frac{1}{\sqrt{{\mu }_{r}{\mu }_{0}\sigma \pi f}},$$

where *f* is the frequency of the present RCL-circuit.

Three different bulk metallic glasses, viz. Cu_46_Zr_46_Al_8_, Cu_44_Zr_44_Al_8_Hf_2_Co_2_, and Zr_52.5_Cu_17.9_Ni_14.6_Al_10_Ti_5_, have been subjected to the flash-annealing. They all have a similar room-temperature resistivity (*ρ* = 1/*σ*) of 1.66 · 10^−6^ Ωm, 1.56 · 10^−6^ Ωm, and 1.61 · 10^−6^ Ωm, respectively. Inserting a relative permeability of *μ*_r_ = 1 and a frequency of 80 kHz into eq. 9, the skin depth amounts to about 2.3 mm for all tested specimens. The as-cast rods have a radius of 2.25 mm, which is slightly less than the calculated skin depth. This estimation already indicates that as-cast samples with a diameter up to 4.5 mm should be heated in a relatively uniform manner. Estimations of Kaltenboeck *et al*.^14^ corroborate this assertion. They have estimated the heating rate in the centre of a Zr-based BMG rod with a diameter of 5 mm and a comparable resistivity of 1.60·10^−6^ Ωm. In their case, heating is ensured by high-frequency induction, so that the central region is heated only due to thermal conductivity. With a thermal diffusivity of 0.03 cm^2^/s (typical of metallic glasses)^14^, they conclude that the centre of the samples is subjected to a maximum heating rate of about 200 K/s regardless of how much faster the surface is heated. This value coincides with the maximum heating rates at the sample surface we can reach with the present flash-annealing setup. And because the radius of all investigated samples is less than *s*, one can reasonably assume that the entire sample volume is heated relatively uniformly during flash-annealing. This is a crucial prerequisite for obtaining uniform composite microstructures through inductive heating.

The next important step after fast heating is quenching, which has to be sufficiently fast in order to preserve the microstructure of the specimen having formed at a given temperature. This is implemented by ejecting the samples into a beaker filled with water. Cooling rates inherent to casting processes can be estimated indirectly from microstructural features such as grain size, dendrite arm spacings or eutectic interlamellar spacings^37^. We used the same approach in the present work and estimated the cooling rate based on the interlamellar spacing, *λ*, of the eutectic alloy Al_82.7_Cu_17.3_^38^. In order to facilitate the transferability of the findings to the BMG specimens, which have a slightly different shape, we only considered Al_82.7_Cu_17.3_ spheres with diameters comparable to those of the rods.

**Figure 2:**
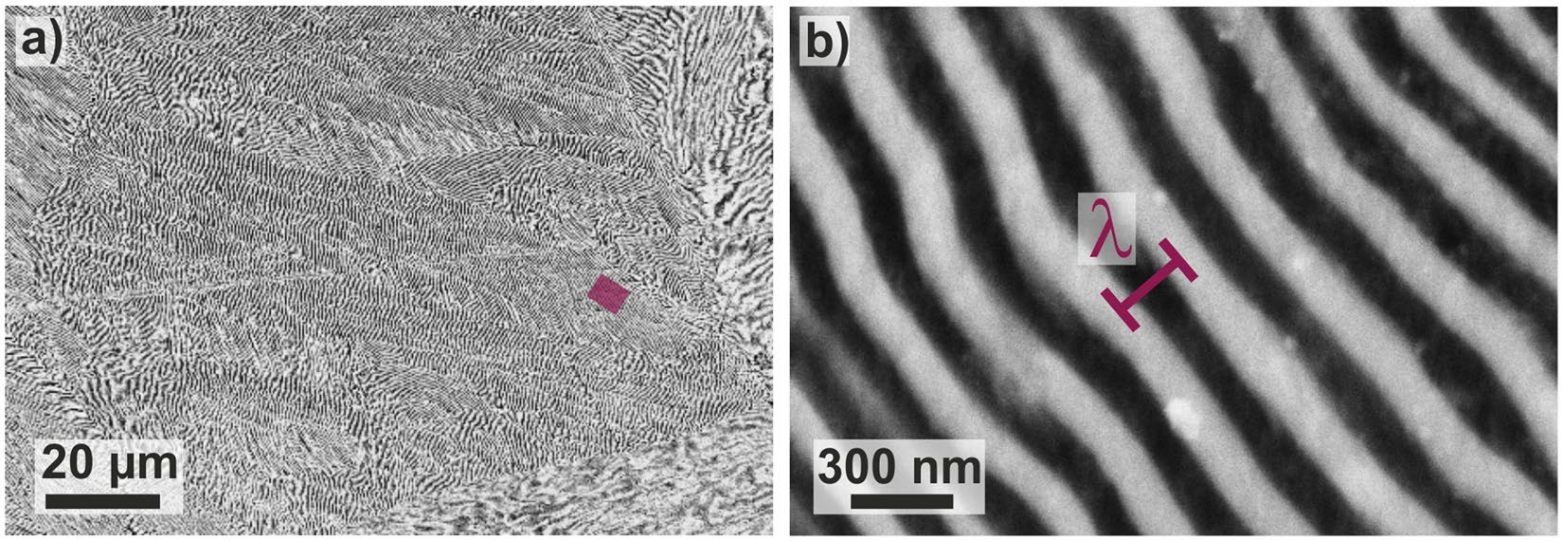
Eutectic microstructure of flash-annealed Al_82.7_Cu_17.3_-specimens. Five independent samples were molten and quenched in water for microstructural investigations. (**a**) SEM-image shows multiple eutectic domains. (**b**) The high-magnification SEM micrograph depicts a eutectic island in which the interlamellar spacing, *λ*, is marked. The magnified area is marked with a purple rectangular in (**a**).

Figure 2a depicts the fine eutectic microstructure in the centre of a solidified Al_82.7_Cu_17.3_ specimen^38^, which consists of several domains. The estimation of the cooling rate is based on the heat balance of the system^38, 39^:

10 $${q}_{e}\frac{A}{V}=-\,\frac{{c}_{p}dT}{dt}+\frac{{\rm{\Delta }}{H}_{f}}{{V}_{m}}\frac{d{f}_{s}}{dt},$$

where *q*_e_ is the external heat flux density at the surface, *A* is the surface area, *V* is the volume, *c*_p_ is the heat capacity, d*T/*d*t* is the cooling rate, *ΔH*_f_ is the heat of fusion, *V*_m_ is the molar volume, and d*x/*d*t* is the change of the crystalline volume fraction with time. We can approximate the slightly irregular-shaped droplets as spheres with a radius, *R*. The solidification starts at the surface of the spherical liquid when it is immersed in water. So, at the onset of solidification, the position of the solid-liquid interface within the sphere, *R*_s_, is given by *R*_s_ = *R*. *f*_s_ represents the solidified volume fraction corresponding to *R*_s_. The freezing rate, d*f*_s_ */*d*t*, and the solidification front velocity d*R*_s_ */*d*t* = *ν* are related according to ref. 38:

11 $$\frac{d{f}_{s}}{dt}=-\,3\nu \frac{{R}_{s}^{2}}{{R}^{3}}$$

As the supercooled liquid solidifies, the temperature is constant (d*T* = 0). Based on eqs (10) and (11), a relationship between the cooling rate, d*T/*d*t*, and the solidification front velocity, *v*, can be established. By means of the Jackson-Hunt relation^40^ eq. (12), one can replace the solidification front velocity by the interlamellar spacing, *λ*. This yields the final eq. (13) between the experimentally determined *λ* and the cooling rate:

12 $${\lambda }^{2}\nu =K,$$

13 $$\frac{dT}{dt}=\frac{{\rm{\Delta }}{H}_{f}}{{V}_{m}}\frac{3K}{{c}_{p}R{\lambda }^{2}}$$

*K* (= 27.5 · 10^−12^ cm^3^/s)^38^ is constant and has been determined from unidirectional solidification experiments of Al_82.7_Cu_17.3_. *ΔH*_*f*_/*(c*_p_ *·V*_m_ *)* (= 440 K*)* ^38^ is constant as well.

The average lamellar spacing, *λ*_ave_, constitutes 93 ± 15 nm and the calculated cooling rate according to (13) is about 7000 ± 1000 K/s. This cooling rate is at least one order of magnitude higher than the highest cooling rate applied in the suction-casting device to obtain the metallic glass in the first step^38^. It exceeds the critical cooling rate of the present alloy^41^, so that the supercooled liquid can be quenched into a glass again^18^. And indeed, water-quenching is sufficient to preserve the microstructure of flash-annealed BMGs and enables the preparation of BMG composites in a controlled fashion as will be shown in the following.

**Figure 3:**
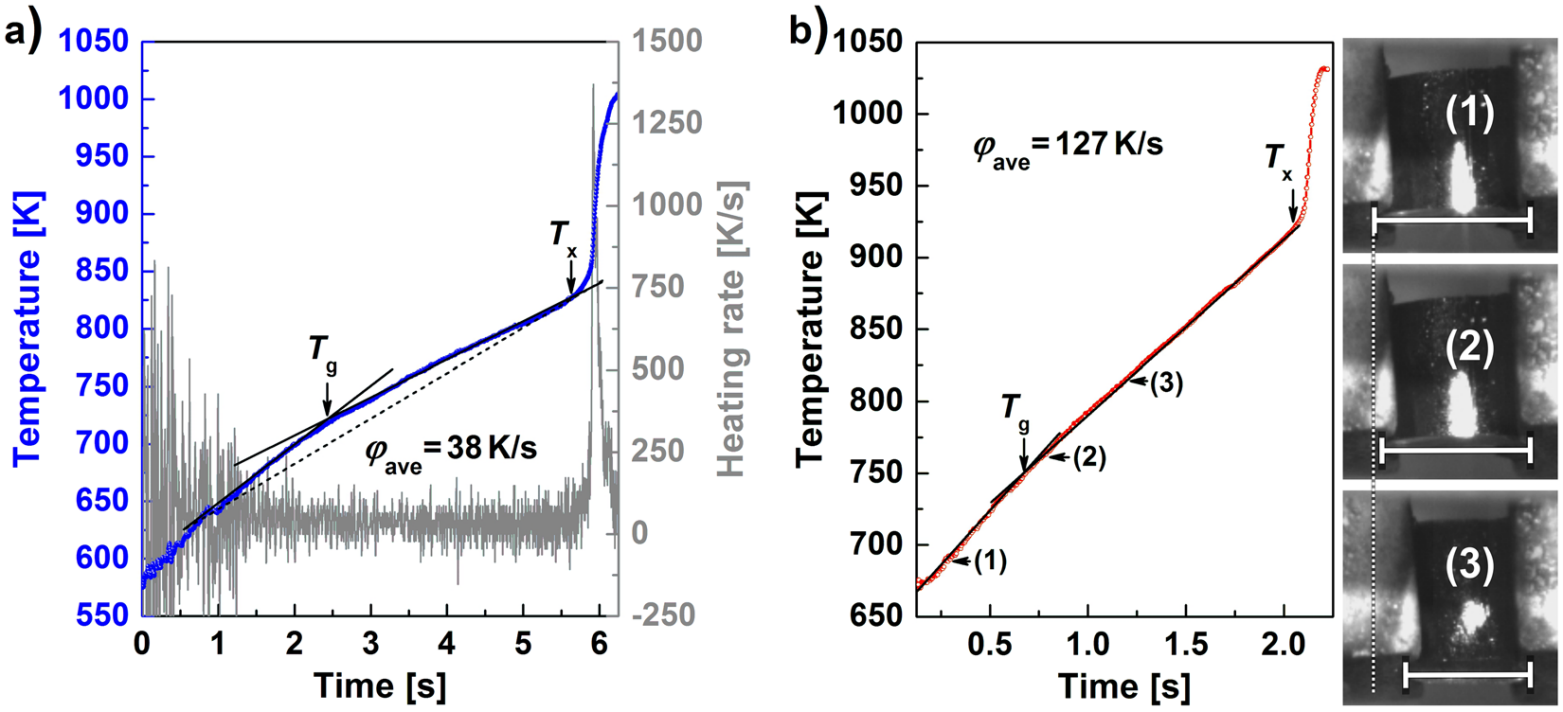
Temperature-time curve of BMG specimens (Ø = 4.5 mm, h = 4 mm) during flash-annealing. (**a**) Representative temperature-time heating curve and its derivative for a Zr_52.5_Cu_17.9_Ni_14.6_Al_10_Ti_5_ BMG specimen flash-annealed at *φ*_ave_ = 38 K/s to *T*_ej_ = 1005 K. The intersection of the two linear fits marks the glass-transition temperature, *T*_g_, and the beginning of the steep temperature increase indicates the crystallization temperature, *T*_*x*_. (**b**) Heating curve of a Cu_44_Zr_44_Al_8_Hf_2_Co_2_ BMG specimen (*φ*_ave_ = 127 K/s) to which a load is applied during heating. Snapshots of a high-speed camera show that the shape of the specimen starts to change at temperatures higher than the intersection. The specimen is deformed more and more and hence the intersection of both linear fits marks *T*_g_.

After the typical heating and cooling characteristics of the present setup are identified, we would like to discuss the behaviour of Cu_46_Zr_46_Al_8_, Cu_44_Zr_44_Al_8_Hf_2_Co_2_, and Zr_52.5_Cu_17.9_Ni_14.6_Al_10_Ti_5_ BMGs flash-annealed under different conditions. By varying the generator power, the BMGs can be heated at a controlled rate to the ejection temperature, *T*_ej_, at which they are released into the water-bath. Figure 3a presents a typical temperature-time (*T*-*t*) curve of glassy Zr_52.5_Cu_17.9_Ni_14.6_Al_10_Ti_5_. The temperature scatters particularly at the beginning due to the sensitivity of the pyrometer. The slope of the heating curve slightly decreases at *T* = 718 K as can be seen from the two linear fits. The resistivity of metallic glasses is slightly higher than that of the supercooled liquid^42, 43^ and, therefore, the intersection of both lines marks the glass-transition temperature, *T*_g_. To substantiate this interpretation, the flash-annealing process was recorded with a high-speed camera. A small pressure is exerted by the clamping system on the sample during heating. And because the viscosity of metallic glasses drops measurably above *T*_g_^44, 45^, it suffices to plastically deform the sample. The camera monitors the shape change during flash-annealing and synchronization of the camera with the pyrometer allows correlating the images with the corresponding temperature. Figure 3b shows the typical heating curve of a Cu_44_Zr_44_Al_8_Hf_2_Co_2_ BMG specimen (*φ*_ave_ = 128 K/s, *T*_ej_ = 1031 K). Also here the two slopes become evident. Snapshots before and after the intersection of the two linear fits (Fig. 3b) reveal that the sample begins to be plastically deformed just above the intersection temperature. Consequently, the slight decrease of the slope in the *T*-*t* curve originates from the glass transition. This furthermore demonstrates that the present setup is in general also suitable for thermoplastically forming BMGs and could be used to modify the surface or the entire sample shape in a fast process with a high throughput.

**Figure 4:**
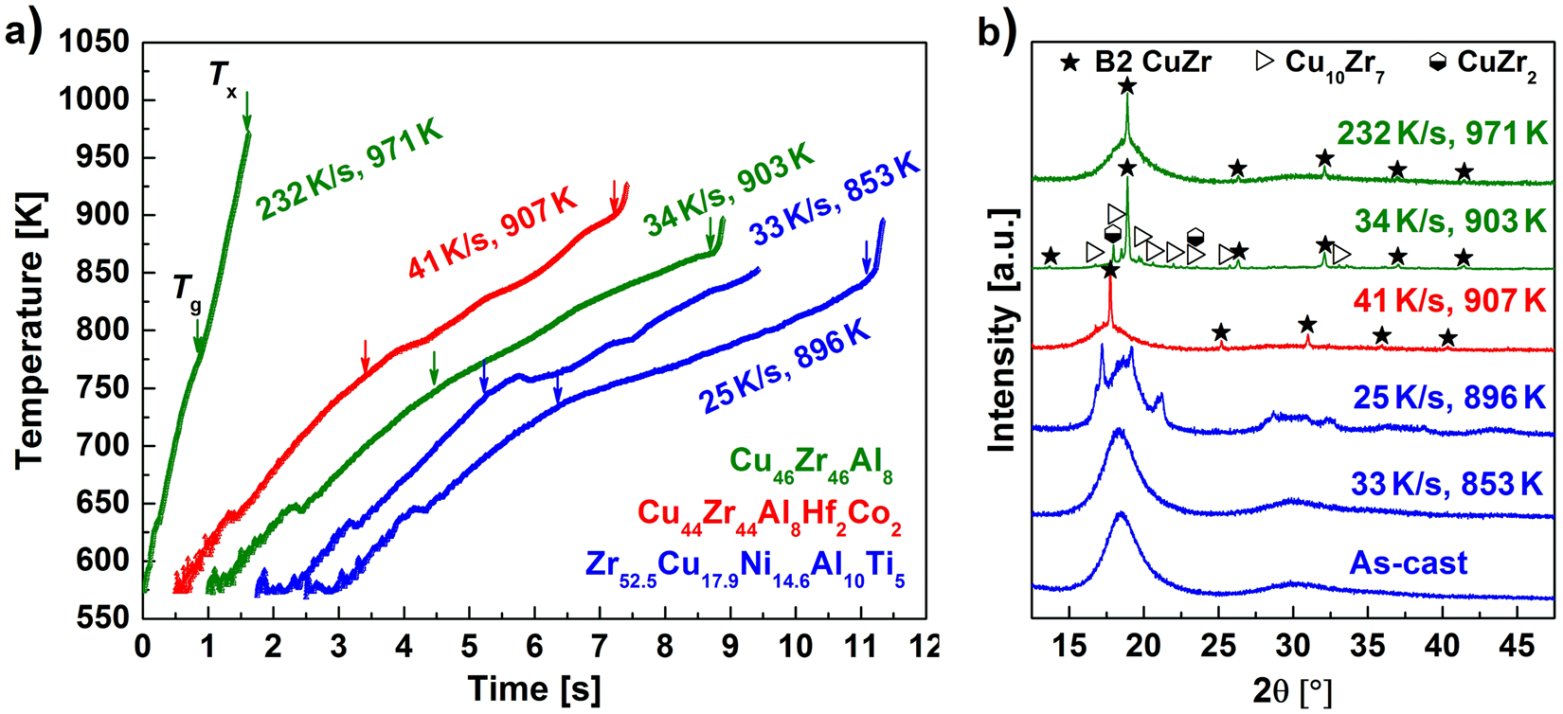
Characteristics of BMG specimens during flash-annealing. (**a**) Heating curves of Cu_46_Zr_46_Al_8_, Cu_44_Zr_44_Al_8_Hf_2_Co_2_ and Zr_52.5_Cu_17.9_Ni_14.6_Al_10_Ti_5_ BMG specimens flash-annealed at indicated heating rates and temperatures (below and above *T*_x_). (**b**) XRD patterns of the flash-annealed and as-cast glasses. The patterns of the specimens flash-annealed above *T*_x_ display sharp reflections originating from crystals. The heating rates, annealing temperatures and crystalline phases are indicated.

To simplify the subsequent discussion, only an average heating rate, *φ*_ave_, is considered for all flash-annealing experiments. After the glass-transition temperature is traversed, continued heating gives rise to a steep temperature increase (Fig. 3a). It is attributed to recalescence and the temperature at the beginning of it then indicates the crystallization onset temperature, *T*_x_ (=826 K) (Fig. 3a): The X-ray diffraction (XRD) pattern of amorphous Zr_52.5_Cu_17.9_Ni_14.6_Al_10_Ti_5_ heated to temperatures below this steep increase (Fig. 4: *T*_ej_ = 853 K) only shows a broad maximum typical of metallic glass. It is similar to the XRD pattern obtained from the same alloy in the as-cast state (Fig. 4b). Once the BMG is heated to temperatures within the supercooled liquid region, it apparently vitrifies again in the course of quenching. However, when the Zr_52.5_Cu_17.9_Ni_14.6_Al_10_Ti_5_ BMG is flash-annealed at a temperature in the middle of the rapid temperature increase (Fig. 4: *T*_ej_ = 896 K), crystalline reflections are present in the XRD pattern. It should be noted that the identification of the crystalline phases is rather difficult due to their small volume fraction and thus is beyond the scope of the present work. The other two glasses, Cu_46_Zr_46_Al_8_ and Cu_44_Zr_44_Al_8_Hf_2_Co_2_, partially crystallize as well when the sudden temperature increase is reached (Fig. 4b). In these cases, the phase identification is not as complex and we elaborate on the crystallization sequence of the Cu_46_Zr_46_Al_8_ glass in the following. According to the Cu-Zr equilibrium phase diagram, the eutectic phases Cu_10_Zr_7_ and CuZr_2_ are stable up to 981 K^46^. Yet, the XRD pattern of the specimen heated at *φ*_ave_ = 34 K/s to *T*_ej_ = 903 K (Fig. 4b) shows crystalline reflections corresponding to the B2 CuZr phase next to the expected Bragg peaks stemming from thermodynamically stable Cu_10_Zr_7_ and CuZr_2_. Yamamoto *et al*.^27^ have devitrified a very similar BMG, viz. Cu_47_Zr_47_Al_6_, at a heating rate of about 8 K/s and have also observed precipitation of the metastable B2 CuZr phase. Because its formation cannot be favoured thermodynamically, kinetic constraints must be responsible for it crystallizing. In contrast to B2 CuZr, which precipitates polymorphically in Cu_46_Zr_46_Al_8_^47^, the low-temperature phases (Cu_10_Zr_7_ and CuZr_2_) undergo a eutectic crystallization which is a typically diffusion-controlled process^48^. The formation of the eutectic phases is associated with a substantial incubation time, as a consequence^49^. When glasses are annealed for such short durations as for the present flash-annealing, atomic diffusion is limited^48^ and B2 CuZr begins to precipitate polymorphically at temperatures where it is still metastable. If the heating rate is sufficiently high, the formation of the low-temperature phases can be completely suppressed and metastable B2 CuZr crystallizes exclusively (Fig. 4b: *φ*_ave_ = 232 K/s, *T*_ej_ = 971 K). These results furthermore confirm our supposition that the cooling rates are sufficient to prevent the eutectoid decomposition of the B2 CuZr phase on quenching. In other words, the microstructures at high temperatures can be preserved indeed.

Interestingly, crystallization of Cu_10_Zr_7_ and CuZr_2_ can be suppressed at much lower heating rates in glassy Cu_44_Zr_44_Al_8_Hf_2_Co_2_. Flash-annealing of Cu_44_Zr_44_Al_8_Hf_2_Co_2_ BMG specimens at *φ*_ave_ = 41 K/s already leads to the sole formation of B2 CuZr (Fig. 4b). By adding minor amounts of Co^50, 51^ to Cu_46_Zr_46_Al_8_, the B2 CuZr phase can be apparently stabilized. The diffusion field in front of the low-temperature phases seems to be more complex when more elements are involved. Hence, the incubation times for both low-temperature phases are longer and their formation can be hampered more efficiently. This quinary alloy might serve as a “model” alloy to study fundamental crystallization processes in highly undercooled melts^18^.

**Figure 5:**
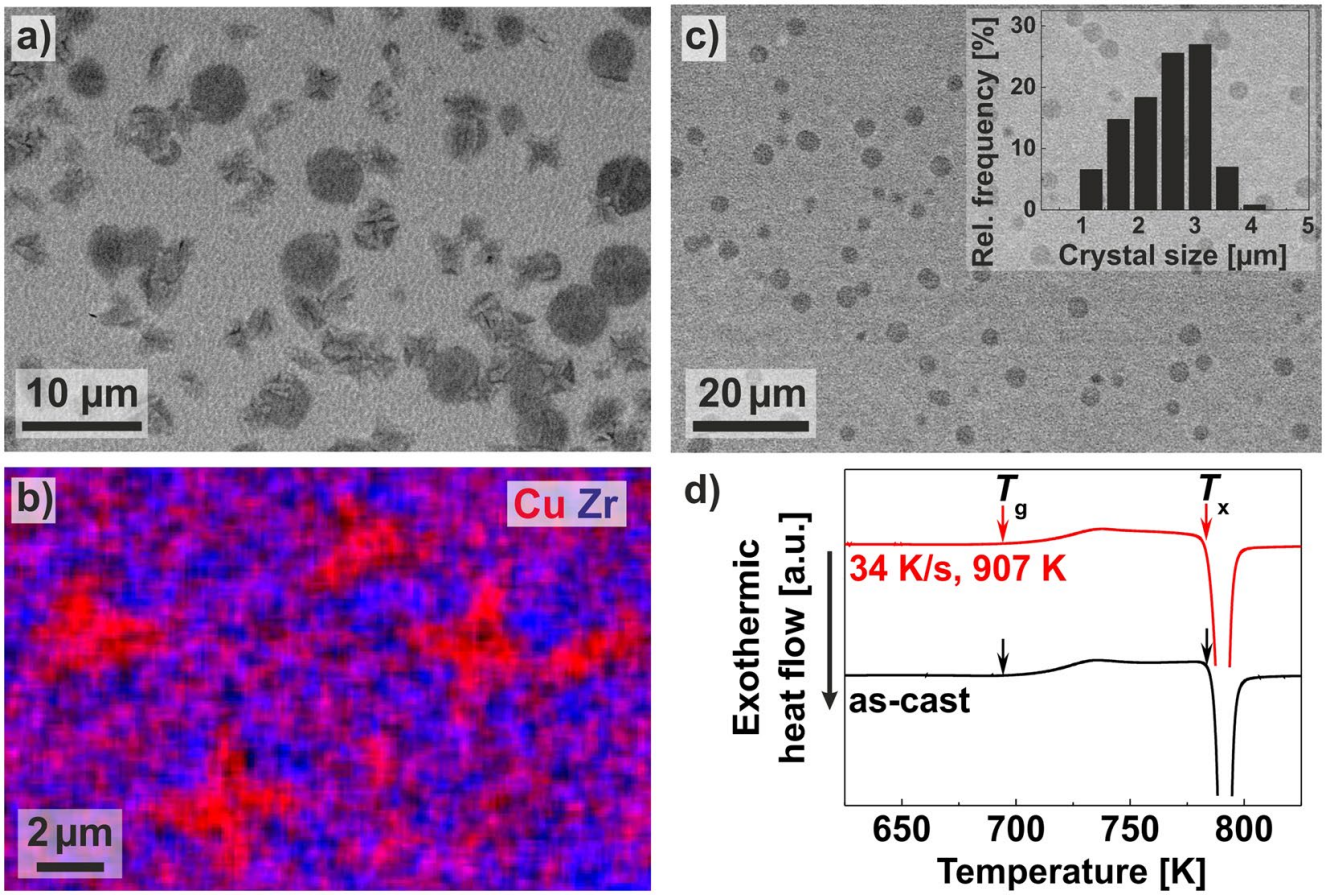
Microstructure of Cu-Zr-based BMGs flash-annealed to temperatures above *T*_x_. (**a**) SEM-image of a Cu_46_Zr_46_Al_8_ BMG flash-annealed at *φ*_ave_ = 34 K/s to *T*_ej_ = 903 K. The microstructure consists of Cu_10_Zr_7_ crystals with a dendritic shape, spherical B2 CuZr particles and a glassy matrix. (**b**) The corresponding EDX map proves the presence of the Cu-rich Cu_10_Zr_7_ dendrites. Their immediate surroundings are enriched in Zr. (**c**) SEM-image near the surface of a glassy Cu_44_Zr_44_Al_8_Hf_2_Co_2_ rod, which was flash-annealed at *φ*_ave_ = 41 K/s to *T*_ej_ = 907 K. Only spherical B2 CuZr particles are embedded in the amorphous matrix and the inset depicts the crystal size distribution. (**d**) DSC curves of the flash-annealed Cu_44_Zr_44_Al_8_Hf_2_Co_2_ specimen and the respective as-cast specimen show the glass transition (*T*_g_) as well as crystallization (*T*_x_).

All XRD patterns of the flash-annealed BMGs reveal a broad maximum typical of metallic glasses on which the sharp, crystalline reflections are superimposed (Fig. 4b). The BMG specimens are only partially devitrified and the microstructure of the obtained BMG-matrix composites will be now addressed more in detail: Fig. 5a depicts a SEM image of the Cu_46_Zr_46_Al_8_ specimen, which was flash-annealed at *φ*_ave_ = 34 K/s to *T*_ej_ = 903 K. Spherical B2 CuZr crystals have nucleated uniformly in the glassy matrix. According to the corresponding XRD pattern (Fig. 4b), Cu_10_Zr_7_ and CuZr_2_ are also present after flash-annealing and, in fact, the SEM image depicts additional particles with a dendritic morphology. These particles are enriched in Cu (red) and Zr (blue) concentrates in their vicinity, as can be seen from the respective energy-dispersive X-ray spectroscopy (EDX) map (Fig. 5b). The Cu-rich particles must hence correspond to Cu_10_Zr_7_ and CuZr_2_ appears to be present in their immediate surroundings. Figure 5c depicts the microstructure of the Cu_44_Zr_44_Al_8_Hf_2_Co_2_ BMG heated at a rate of *φ*_ave_ = 41 K/s to an ejection temperature of *T*_ej_ = 907 K. As the XRD pattern (Fig. 4b) suggests, the microstructure should be only comprised of B2 CuZr crystals and glass. Indeed, only finely dispersed spherical B2 CuZr crystals are embedded in a featureless amorphous matrix (Fig. 5c). In order to get a more quantitative description of the composite microstructures, the crystal sizes were statistically analyzed. The respective crystal size distribution (CSD) is extremely narrow (Fig. 5c, inset) and the average crystal size of 4 ± 2 µm is between one to two orders of magnitude smaller than the typical size of B2 CuZr crystals in CuZr-based BMG composites prepared by casting^22–25, 52^. As mentioned above, this is caused by the short annealing time and the fact that the maximum growth rate is not reached during flash-annealing.

As demonstrated above, the glassy phase can be thermoplastically formed at temperatures just above the glass-transition temperature (Fig. 3b). The present setup, consequently, allows processing these uniform BMG-matrix composites by thermoplastic forming into net-shape geometries^53^ in a second flash-anneal at lower temperatures within the same device. This is considered to be of technological interest because in this way small high-strength components with an increased plastic deformability can be processed quickly and rather effortlessly.

Figure 5d discloses the DSC traces of the as-cast and flash-annealed Cu_44_Zr_44_Al_8_Hf_2_Co_2_ BMGs. The traces show a glass transition marked by *T*_g_ and a crystallization event, whose beginning is marked as *T*_x_. Both events confirm the presence of the glass in the flash-annealed BMG (Fig. 5d: *φ*_ave_ = 34 K/s, *T*_ej_ = 907 K) and corroborate the XRD results (Fig. 4b). Due to partial crystallization of the B2 CuZr phase, the crystallization enthalpy, *ΔH*_x_, is lower for the flash-annealed (*ΔH*_x_ = 54 ± 1 J/g) than for the as-cast BMG (*ΔH*_x_ = 57 ± 1 J/g).

**Figure 6:**
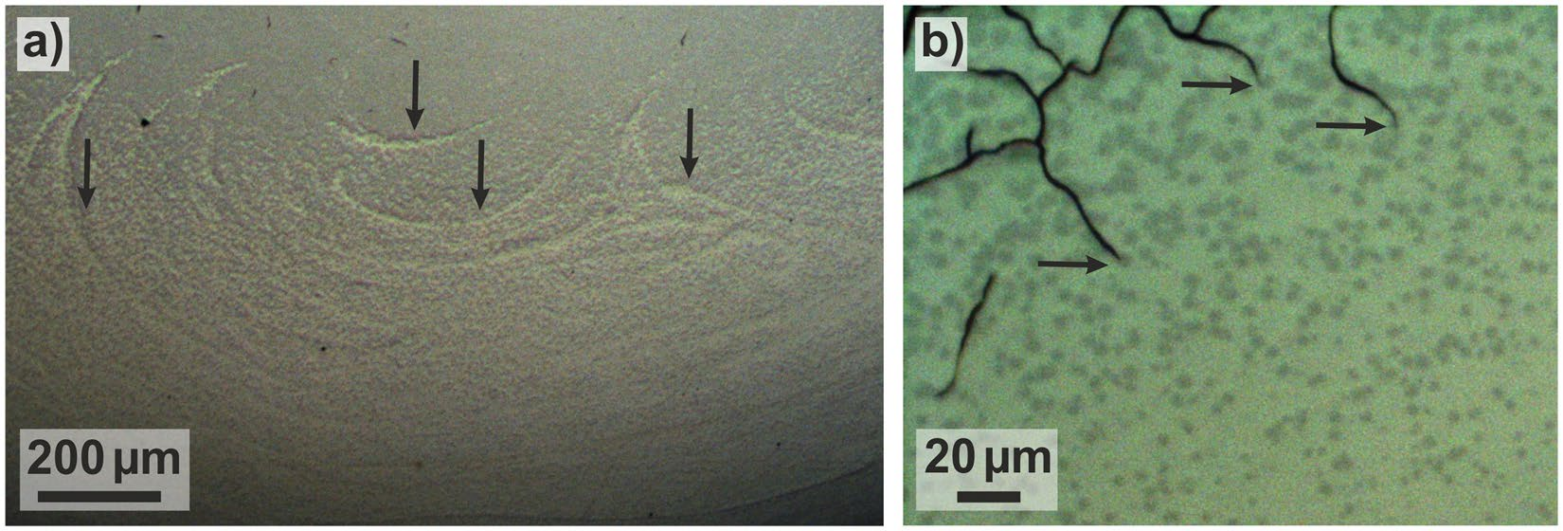
Partially crystalline microstructure of a Zr_52.5_Cu_17.9_Ni_14.6_Al_10_Ti_5_ BMG flash-annealed at *φ*_ave_ = 37 K/s to *T*_ej_ = 878 K. (**a**) Optical micrograph (OM) of a BMG heated. The crystals are preferentially aligned along the eddy currents (brighter curved traces marked with vertical arrows). (**b**) OM of the same BMG specimen depicts cracks, which form and propagate in the crystalline phase network and terminate in the amorphous matrix.

After partial devitrification of the Zr_52.5_Cu_17.9_Ni_14.6_Al_10_Ti_5_ BMG, one can observe a pattern of crystals, which indicates the flow of the eddy currents in the sample during inductive heating (Fig. 6a: vertical black arrows). After the BMG is heated to above *T*_g_, eddy currents seem to stir the supercooled liquid and crystals preferentially form along these eddy currents^35, 36^. In contrast to the relatively soft and ductile B2 CuZr phase^25, 54^ in the other two glasses, the crystalline decomposition products in Zr_52.5_Cu_17.9_Ni_14.6_Al_10_Ti_5_ are very brittle^55^. This manifests in a high density of cracks caused by cooling-induced thermal stresses. The cracks propagate within the brittle crystalline network^56^ and terminate in the amorphous matrix (Fig. 6b). Consequently, the amorphous phase of flash-annealed BMGs appears to be tougher than the crystalline precipitates. This demonstrates the importance of identifying glass-forming systems in which the formation of a soft and ductile stable or metastable crystalline phase is favoured during crystallization far from equilibrium.

**Figure 7:**
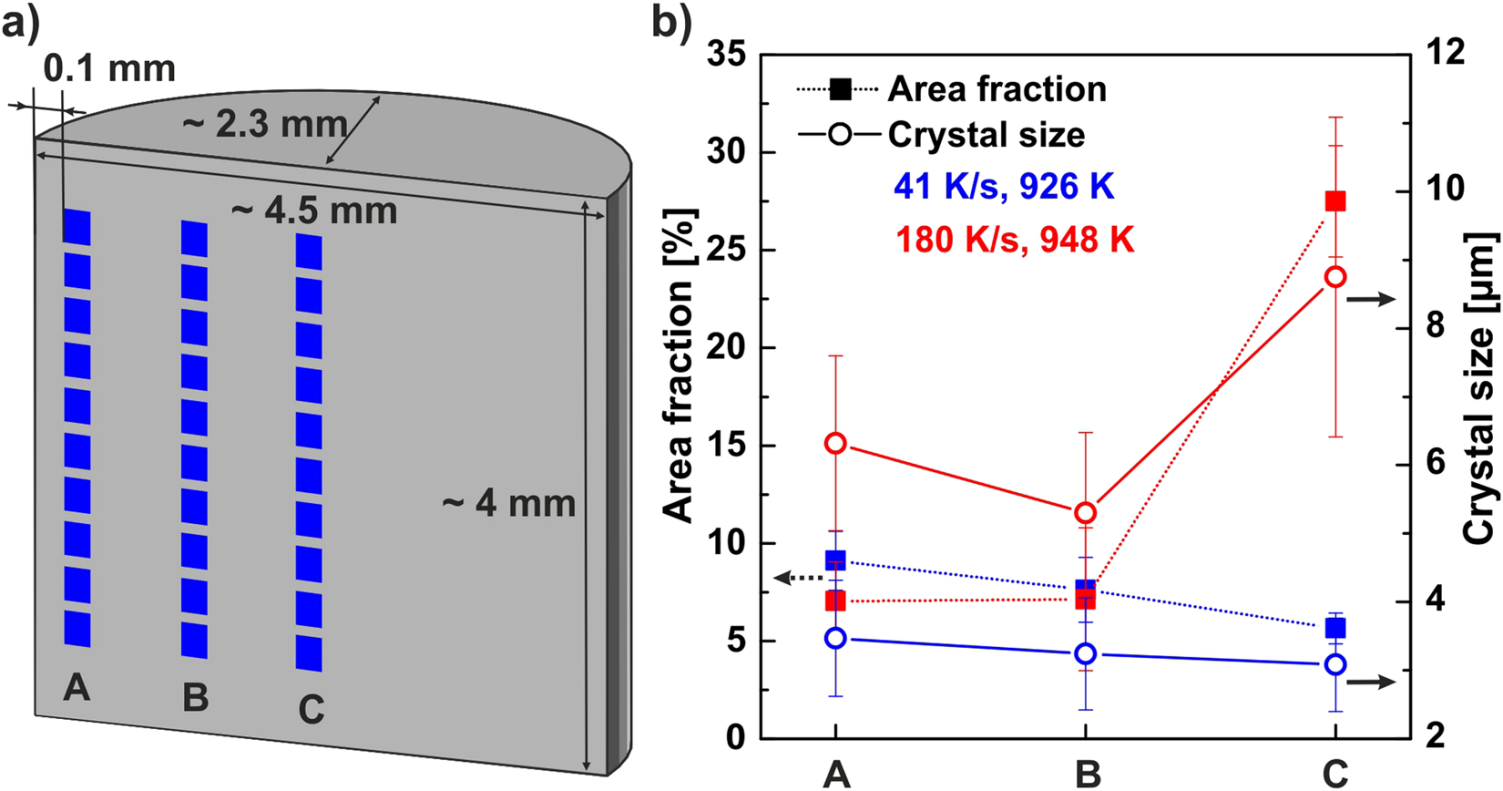
Influence of the heating rate on the distribution and size of B2 CuZr crystals in flash-annealed Cu_44_Zr_44_Al_8_Hf_2_Co_2_ BMGs. (**a**) Sketch of how the microstructure was analyzed. Ten SEM-images at different magnifications were taken and processed from each section (A, B and C). (**b**) Area fraction and particle size of B2 CuZr crystals as a function of the location along the longitudinal section.

In the next step, the distribution of crystals across the samples was analyzed. Due to thermal gradients on heating and cooling one might expect that the crystals are not uniformly distributed in the glassy matrix. Therefore, the longitudinal sections of two Cu_44_Zr_44_Al_8_Hf_2_Co_2_ BMG-matrix composites annealed under different conditions (*φ*_ave_ = 41 K/s and 181 K/s, *T*_ej_ = 926 K and 948 K) were divided into three regions: A, B and C (Fig. 7a) and the crystal size distributions systematically analyzed. The results shown in Fig. 7b and Table 1 are based on ten independent micrographs of each section.

**Table 1:** Quantitative analysis of the microstructure of Cu_44_Zr_44_Al_8_Hf_2_Co_2_ BMG composites containing B2 CuZr crystals and a glassy matrix.

*φ*	*T*_ej_	Area fraction			Crystal size		
[K/s]	[K]	[%]			[µm]		
		A	B	C	A	B	C
41 ± 5	926 ± 5	9 ± 1.5	8 ± 1.7	6 ± 0.8	3.5 ± 0.85	3.3 ± 0.82	3.1 ± 0.68
180 ± 10	948 ± 5	7 ± 2.0	7 ± 3.7	28 ± 2.8	6.3 ± 1.28	5.3 ± 1.18	8.8 ± 2.34

The area fraction and size of the B2 CuZr crystals are listed to indicate their dependence on the heating rate and location within the specimen. The average heating rate, *φ*_ave_, and ejection temperature, *T*_ej_, are given additionally.

For the specimen heated at *φ*_ave_ = 41 K/s to *T*_ej_ = 926 K, the B2 CuZr crystals cover an area fraction of 9 ± 1.5%, 8 ± 1.7%, and 6 ± 0.8% in the region A, B, and C, respectively (Fig. 7b and Table 1). The area fraction decreases slightly from the A- to the C-region of the specimen. This trend can be ascribed to the skin effect, which heats the region near the lateral surface faster. The temperature near the surface reaches *T*_x_ after a shorter time than the centre of the specimen. Hence, B2 CuZr crystals start to nucleate earlier. The crystal size shows the same trend. It decreases from 3.5 ± 0.85 µm at the A-region to 3.3 ± 0.82 µm at the B-region to 3.1 ± 0.68 µm at the C-region of the specimen (Fig. 7b and Table 1). BMG specimens are generally heated and cooled faster near the surface than in their interior. Nonetheless, these gradients do not result in significantly more or larger crystals. On the contrary, these temperature gradients seem to effectively compensate their effects on the microstructure at this heating rate and ejection temperature. In order to determine whether the uniformity of the composite microstructure depends on the heating rate, another Cu_44_Zr_44_Al_8_Hf_2_Co_2_ BMG sample was flash-annealed at *φ*_ave_ = 181 K/s to *T*_ej_ = 948 K. The area fraction of the B2 CuZr phase is constant in the region A (7 ± 2.0%) and B (7 ± 3.7), but increases drastically in the sample centre, where 28 ± 2.8% of the area constitute crystals (Fig. 7b and Table 1). The gradient in the cooling rate in combination with the faster release of latent heat at higher heating rates keeps the centre of the sample at elevated temperatures for longer times and stimulates the precipitation of crystals. The faster the BMG is flash-annealed, the faster release of latent heat, the less uniform is the microstructure of the BMG-matrix composite. Compared to BMG-matrix composites prepared by melt-quenching, the distribution of crystals and their size is remarkably uniform. Except of a tedious re-melting treatment^30^, no other procedure is capable of preparing such uniform BMG-matrix composites to date.

It shall be stressed here that the volume fraction as well as the crystal size can be controlled almost independently during flash-annealing by modifying the heating rate and the ejection temperature^18^. Therefore, also the deformation behaviour can be altered and adjusted to a large extent. This helps in revealing the detailed interrelations between the microstructure and the mechanical properties on one side and the deformation mechanisms on the other. In this respect, flash-annealing opens up new possibilities in the synthesis of BMG-matrix composites and bears the potential with regard to designing their microstructures as well as their mechanical properties.

## Summary and Conclusions

An inductive flash-annealing device was developed and Cu_46_Zr_46_Al_8_, Cu_44_Zr_44_Al_8_Hf_2_Co_2_ and Zr_52.5_Cu_17.9_Ni_14.6_Al_10_Ti_5_ BMGs were flash-annealed to given temperatures followed by immediate quenching in a water-bath. These glasses can be heated in a temperature-controlled manner with rates of up to about 200 K/s. The effective cooling rate, which was estimated from solidification experiments of the eutectic Al_82.7_Cu_17.3_ alloy, is of the order of 10^3^ K/s and enables preservation of the microstructure, which has formed during flash-annealing. The present setup allows recording the temperatures during heating and the glass-transition temperature, *T*_g_, as well as the onset of crystallization, *T*_x_, can be identified. Because both shift systematically with the heating rate, detailed continuous heating transformation (CHT) diagrams can be constructed^18^.

Cu_46_Zr_46_Al_8_, Cu_44_Zr_44_Al_8_Hf_2_Co_2_ and Zr_52.5_Cu_17.9_Ni_14.6_Al_10_Ti_5_ BMGs were flash-annealed to temperatures below and above *T*_x_. Once the BMGs are heated to temperatures within the supercooled liquid region (*T*_g_ ≤ *T* ≤ *T*_x_), the inherently high cooling rates of our flash-annealing device allow for re-vitrification. If the metallic glasses are flash-annealed to temperatures above *T*_x_, BMG-matrix composites can be prepared. The phase evolution depends on the alloy composition and the heating rate. High rates impose kinetic constraints on the crystalline phase, which precipitates in the supercooled liquid. For CuZr-based BMGs, the crystallization process changes from eutectic (Cu_10_Zr_7_ and CuZr_2_) to polymorphic (B2 CuZr) above a critical heating rate. The present approach paves the way for investigating the effect of kinetic constraints on the phase evolution in a multitude of different glass-forming alloys, which may be accompanied by interesting mechanical properties.

The microstructure of these BMG-matrix composites is comprised of small (up to 10 µm) crystals being finely dispersed in the glassy matrix. Their average size is at least one order of magnitude smaller than the size of B2 CuZr crystals of BMG-matrix composites prepared by casting. The distribution of the crystalline phase within the BMG-matrix composites is remarkably uniform. Flash-annealing of BMGs, hence, represents a new approach to design the BMG-matrix composite microstructures and with it to optimize their mechanical properties. Even though the sample diameter is limited to about 4.5 mm and inductive flash-annealing cannot be scaled because of the inherent skin effect, sufficiently large specimens can be synthesized in order to address fundamental scientific questions related to the phase evolution and deformation mechanisms in BMG-matrix composites.

## Methods

Pre-alloys of Zr_52.5_Cu_17.9_Ni_14.6_Al_10_Ti_5_, Cu_46_Zr_46_Al_8_ and Cu_44_Zr_44_Al_8_Hf_2_Co_2_ were prepared from high-purity elements (purity 99.99%) by arc-melting in a Ti-gettered Ar-atmosphere. These ingots were remolten three times to ensure chemical homogeneity. Pieces of the ingots with an approximate weight of 7 g were used to fabricate 50 mm long rods with a diameter of 4.5 mm via suction casting into a water-cooled copper mould. Afterwards they were sliced into 4 mm long pieces by means of a Struers Accutom-50. The Al_82.7_Cu_17.3_ pre-alloy was prepared by centrifugal casting.

The flash-annealing device was developed at the IFW Dresden and consists of a generator (10 kW, Trumpf Hüttinger), an in-house designed working coil and clamping system with an argon-shower, a control unit equipped with a Siemens Simatic PLC and a Lumasense ms-pyromter, which measures from 573 to 1273 K. The surface temperature of the specimen, which is heated within the working coil, is monitored by the ms-pyrometer. When a preset temperature is reached, the control unit activates the clamping system and the sample falls into a subjacent water-bath. The surface temperature obtained from the pyrometer was double-checked with a thermocouple (type T). The high-speed recording was performed by means of a Photron Fastcam SA3.

The as-cast and flash-annealed specimens were characterized by X-ray diffraction in transmission mode (STOE STADI P) with Mo-K_α1_ radiation (*λ*_Mo_ = 0.07093187 nm). The resistivity of the as-cast bulk metallic glasses was determined at room temperature by means of a Quantum Design PPMS Model 6000 equipped with a AC transport controller 7100. Microstructural investigations were carried out with a Nikon Epiphot 300 optical microscope and a Zeiss Gemini 1530 electron microscope equipped with a Bruker Xflash 4010 spectrometer to conduct energy-dispersive X-ray spectroscopy (EDX). The micrographs were evaluated with the program “Leica QWin” to obtain the area fraction and particle size of the crystalline phase. The interlamellar distances of the Al_82.7_Cu_17.3_ eutectic structure were determined using the software “ImageJ”. Thereby, eutectic specimens were molten within the coil and dropped into a subjacent water-bath. Only the eutectic domains with the smallest interlamellar spacing, *λ*, were considered to ensure that only domains, which are perpendicular to the image plane are considered. Five independent specimens with a typical diameter of 4–5 mm were analyzed. SEM-images of ten domains of the centre of each specimen were considered. The interlamellar spacing was determined from eutectic domains, which consist of at least twelve lamellae.
